# Exploring Social Media Preferences for Healthy Weight Management Interventions Among Adolescents of Color: Mixed Methods Study

**DOI:** 10.2196/43961

**Published:** 2023-05-08

**Authors:** Selenne Alatorre, Aviva G Schwarz, Kelsey A Egan, Amanda R Feldman, Marielis Rosa, Monica L Wang

**Affiliations:** 1 Department of Community Health Sciences Boston University School of Public Health Boston, MA United States; 2 Division of General Academic Pediatrics, Department of Pediatrics Boston Medical Center, Boston University Chobanian & Avedisian School of Medicine Boston, MA United States; 3 College of Arts and Sciences Boston University Boston, MA United States; 4 Office of Narrative Boston University Center for Antiracist Research Boston, MA United States; 5 Department of Health Policy and Management Harvard T.H. Chan School of Public Health Boston, MA United States

**Keywords:** social media, adolescents of color, obesity disparities, disparity, disparities, healthy weight management, health education, child health, mHealth, mobile health, weight, obese, obesity, child, pediatric, adolescent, adolescence, preference, health behavior, mobile phone

## Abstract

**Background:**

Social media holds promise as an intervention platform to engage youths in healthy weight management and target racial inequities in obesity.

**Objective:**

This mixed methods study aimed to examine social media habits, preferences, and obesity-related behaviors (eg, diet and physical activity) among adolescents of color and understand preferences for healthy weight management interventions delivered via social media.

**Methods:**

This mixed methods study is comprised of a cross-sectional web-based survey and a series of digital focus groups. Study participants (English-speaking youths of color ages 14-18 years) were recruited from high schools and youth-based community settings in Massachusetts and California. For surveys, participants were invited to complete an anonymous web-based survey assessing self-reported sociodemographics, social media habits and preferences, health behaviors (diet, physical activity, sleep, and screen time), and height and weight. For focus groups, participants were invited to participate in 45- to 60-minute web-based group discussions assessing social media habits, preferred social media platforms, and preferences for physical activity and nutrition intervention content and delivery. Survey data were analyzed descriptively; focus group transcripts were analyzed using a directed content analysis approach.

**Results:**

A total of 101 adolescents completed the survey and 20 adolescents participated in a total of 3 focus groups. Participants reported most frequently using TikTok, followed by Instagram, Snapchat, and Twitter; preference for platform varied by purpose of use (eg, content consumption, connection, or communication). TikTok emerged as the platform of choice as an engaging way to learn about various topics, including desired health information on physical fitness and diet.

**Conclusions:**

Findings from this study suggest that social media platforms can be an engaging way to reach adolescents of color. Data will inform future social media–based interventions to engage adolescents of color in healthy weight management content.

## Introduction

The high rates of adolescent obesity in the United States remain a significant public health concern. Nearly 1 in 4 (22.2%) adolescents aged 12-19 years in the United States had obesity from 2017 to 2020 [1]. Youths of color experience disproportionately higher rates of obesity, with 26.2% of Hispanic and 24.8% of non-Hispanic Black youths ages 2-19 years with obesity, compared to 16.6% of their non-Hispanic White peers [1]. Given the adverse effects associated with adolescent obesity, including elevated risk for diabetes, heart disease, and shorter life expectancies, developing intervention strategies with a health equity lens to prevent and treat obesity, particularly among youths of color who face disproportionately higher rates, is critical [2-4].

Digital or mobile health interventions, defined as health services delivered electronically through formal or informal care, hold promise as a modality to engage adolescents in improving obesity-related health behaviors, such as diet and physical activity [5-8]. Studies to date that have evaluated digital health interventions as a primary or supplemental tool within behavior change obesity interventions have primarily targeted adults or have assessed programs that jointly engage adolescents and their parents or guardians [6,9,10]. Prior digital health interventions targeting adolescents have largely focused on chronic disease management (eg, type 1 diabetes) rather than health behavior change related to obesity prevention and treatment [7,11]. Further, research on digital health interventions targeting obesity-related behaviors with content and strategies tailored for adolescents of color is limited.

Given the high prevalence of smartphone and social media use among adolescents, social media–delivered health interventions are a promising approach to engage adolescents in healthy weight management [12]. In 2021, 84% of adolescents 13-18 years of age reported ever using social media [13]. Social media use among this age group increased during the COVID-19 pandemic, with an average of 1 hour and 27 minutes per day spent on social media in 2021, up from 1 hour and 10 minutes in 2019 [13]. A 2018 Pew Research study found that 95% of 13- to 17-year-olds reported having access to a smartphone and nearly half reported being on the internet almost constantly, with over 70% of Black and Latine youth participants reported using at least 1 social media platform [14]. The Pew study also reported gender differences in social media platform preference among adolescents, with females more likely to prefer Snapchat than males and males more likely to prefer YouTube than females. The prevalent use of social media platforms among all adolescents, including those of color, highlights the potential of social media as a vehicle to deliver accurate and engaging health content to promote healthy weight management behaviors.

The limited literature on adolescents’ exposure to and consumption of health information, particularly related to healthy weight management, through social media platforms has produced mixed findings. A small pilot study evaluating the consumption of health- and fitness-related social media content among adolescent females found that participants did not follow health-related pages or accounts and did not actively search for health content on social media [15]. A systematic review of social media interventions with content targeting nutrition and obesity among adolescents and young adults found that two-thirds of such interventions were associated with at least 1 clinical nutritional or dietary behavioral improvement [16]. However, none to our knowledge have specifically examined the efficacy of such interventions among adolescents of color.

This mixed methods study aims to (1) examine social media habits, preferences, and obesity-related behaviors (eg, diet and physical activity) among adolescents of color and (2) understand intervention preferences to inform the development of future healthy weight management behavioral intervention delivered via social media through cross-sectional surveys and focus groups among a sample of adolescents of color.

## Methods

### Design

This study collected quantitative and qualitative data from high school students aged 14-18 years who identified as people of color in order to assess adolescents’ weight management behaviors, attitudes, practices, and social media preferences. Participants were asked to answer questions related to their social media habits, preferences for physical activity and nutrition intervention content and delivery, preferred social media platform, self-reported measures of obesity-related behaviors (eg, diet and physical activity), and height and weight via an anonymous cross-sectional web-based survey and web-based focus groups.

### Ethics Approval

Study procedures were approved by the Institutional Review Board of Boston University Medical Campus (IRB # H-40968).

### Recruitment, Setting, and Procedures

The investigative team contacted youth-based or youth-affiliated organizations in their network, including the Massachusetts Alliance of Boys & Girls Clubs (BGC), high schools in California, and community partners in Massachusetts and California to recruit participants. BGC staff, high school administrators, and community partners in Massachusetts and California were informed of the purpose and methods of the study. In total, 3 BGCs in Massachusetts and 2 high schools in southern California indicated interest in participating and were asked to share physical recruitment flyers with adolescents of color ages 14-18 years. Adolescent participants were also invited to share the survey with their social networks. Survey recruitment and administration took place from March 2021 to April 2022 with interested and eligible participants provided with a web-based QR code to access and complete the 1-time web-based survey. Focus group recruitment and facilitation took place from April 2021 to September 2021. Study staff contacted interested and eligible focus group participants to identify a date and time for a web-based 1-hour meeting (6-8 participants per group). Focus groups were conducted by a trained facilitator using Zoom, lasted 45-60 minutes in duration, and were recorded and transcribed.

### Participants

Inclusion criteria for survey and focus group participants included 14-18 years of age; self-identification as a person of color (African American or Black, Latine or Hispanic, Native American or American Indian, Native Hawaiian or Pacific Islander, Asian, other, or Multiracial); and able to read and communicate verbally in English. Survey participants were asked to provide electronic consent. Focus group participants were recruited from the sample of survey participants. Focus group members provided verbal consent. Participants were offered a US $25 gift card for participating in the study survey and US $50 for participating in the study focus group.

### Measures

#### Survey Measures

Web-based survey questions measured self-reported sociodemographics, health behaviors, height and weight, and social media habits and preferences. Sociodemographics included gender (male, female, or nonbinary); age (years); grade level; Hispanic or Latine ethnicity; race (White, Black or African American, Asian, American Indian or Alaskan Native, Native Hawaiian or Pacific Islander, other, or multiracial) with more than one response permitted; and state or territory of residence in the United States. Items from the 2019 Youth Risk Behavior Surveillance Survey were used to assess health behaviors, including number of servings of fruit, vegetables, and sugar-sweetened beverages (SSBs) on a typical day in the past week [17]; number of days engaged in at least 60 minutes of physical activity per day over the past week; number of hours slept on an average school day and a weekend day over the past week; and number of hours engaged in screen time (nonschoolwork) on an average school day.

Survey items on social media habits and preferences assessed current use of major platforms (TikTok, Instagram, Twitter, Facebook, Snapchat, YouTube, Tumblr, Reddit, and Pinterest) as well as asked participant to identify their single most-used platform. Participants reported usage frequency (several times a day, once a day, 3-5 times per week, 1-2 times per week, every few weeks, and less often) for each platform of interest and were asked to identify their preferred platform to receive health information and connect with other adolescents of color on health behaviors.

#### Focus Group Measures

A trained facilitator asked a series of open-ended questions assessing social media usage and preferences, health-related concerns, and design and content preferences for a social media intervention to address obesity risk behaviors (see Multimedia Appendix 1 for guide).

### Data Analysis

#### Quantitative Survey Data Analysis

Descriptive statistics were compiled to characterize the study population according to sociodemographics, health behaviors, social media usage, and age- and gender-adjusted BMI-based weight status categories. Race and Hispanic or Latine ethnicity were combined into mutually exclusive race and ethnicity categories: Hispanic or Latine; non-Hispanic Black; non-Hispanic Asian; or non-Hispanic other or multiple races. BMI was computed from self-reported height and weight and classified based on age- and gender-specific percentiles as follows: underweight (<5th percentile), healthy weight (5th-84th percentile), overweight (85th-94th percentile), and obese (>95th percentile), with missing or implausible responses excluded. Average daily screen time combined the number of hours watching television and using video games and computers.

For participants who did not respond to the question on single most-used social media platform (n=23), a most-used platform was imputed based on their reported usage frequency of each platform, resulting in 93 responses for this measure. Overall social media usage frequency (several times a day, once per day, or less than once per day) combined the reported frequency of use for each individual platform and was defined as frequent usage if the frequency exceeded once per day.

Differences in health behaviors, social media habits, and covariates of interest by gender (male vs female) and weight status (underweight or healthy weight vs overweight or obese) were explored using chi-square tests. Data presented in the tables were not stratified by gender or weight status as the study was not powered to detect differences by these characteristics, and data were not compared by race or ethnicity, state of residence, or social media usage frequency because the sample lacked sufficient distribution across subgroups. All analyses were conducted using SAS (version 9.4; SAS Institute).

#### Focus Group Data Analysis

Focus group audio was transcribed verbatim and thematically analyzed by study staff. The analysis used a directed content analysis approach, where common themes were identified by a study staff member and used to formulate an initial codebook [18]. Two independent coders reviewed the transcripts and codes and revised the codebook to incorporate additional themes as needed. Initial interrater agreement of coding was 85.7%; discrepancies were resolved via discussion until 100% consensus was reached. The 2 coders also identified quotes that represented themes from focus group discussions.

## Results

### Survey Results

A total of 101 adolescents of color (mean age of 16.4, SD 1.3 years) completed the survey. An equal proportion (48.5%) identified as male and female, and 3.0% identified as nonbinary. The majority of respondents indicated Hispanic or Latine ethnicity (79.0%) and California residence (63.4%). Over half of the participants (53.6%) had BMI in the healthy weight category, followed by overweight (25.8%) and obese (17.5%). Social media usage frequency exceeded once per day for 88.1% of survey participants (see Table 1 for additional characteristics). The top most-used social media platforms included TikTok (40.9%), Instagram (17.2%), Snapchat (11.8%), and Twitter (8.6%; Table 2). The majority of survey participants reported some use of TikTok (80.2%) and Instagram (71.3%). TikTok was also the most frequent response for preferred platform to receive health advice (55.4%) and connect with others (44.0%).

**Table 1:** Characteristics of 101 adolescents of color in a mixed methods social media study (2021).

Characteristics		Values
**Gender, n (%)**		
	Male	49 (48.5)
	Female	49 (48.5)
	Nonbinary	3 (3.0)
Age, mean (SD)		16.4 (1.3)
**Race and ethnicity^a^, n (%)**		
	Hispanic or Latine	79 (79.0)
	Non-Hispanic Black or African American	12 (12.0)
	Non-Hispanic Asian	3 (3.0)
	Non-Hispanic other or multiple races	6 (6.0)
**State of residence^b^, n (%)**		
	California	63 (63.4)
	Massachusetts	28 (28.3)
	Other states	8 (8.1)
**Social media usage frequency, n (%)**		
	Once per day or more	89 (88.1)
	Less than once per day	8 (7.9)
**BMI^c^, mean (SD)**		24.6 (5.2)
	Underweight or healthy, n (%)	55 (56.7)
	Overweight, n (%)	25 (25.8)
	Obese, n (%)	17 (17.5)
**Health behaviors**		
	Number of days engaged in moderate to vigorous physical activity at least 60 minutes per day over the past 7 days, mean (SD)	4.1 (2.2)
	Engaged in daily moderate to vigorous physical activity at least 60 minutes per day, n (%)	27 (26.7)
	Number of fruit and vegetable servings consumed per day, mean (SD)	4.3 (2.5)
	Consumed at least 5 servings of fruits and vegetables on a typical day over the past 7 days, n (%)	44 (43.6)
	Number of sugar-sweetened beverage servings consumed per day over past 7 days, n (%)	1.8 (1.4)
	Consumed 0 or 1 servings of sugar-sweetened beverages per day on a typical day over the past 7 days, n (%)	54 (53.5)
	Screen time (hours per day) over the past 7 days, mean (SD)	4.4 (2.8)
	Hours slept per night on a typical school night over the past 7 days, mean (SD)	7.2 (1.3)
	Slept at least 8 hours per night on a typical school night over the past 7 days, n (%)	39 (38.6)

^a^Race or ethnicity information was missing for 1 respondent.

^b^State of residence was missing for 2 respondents.

^c^BMI was calculated based on self-reported height and weight. Height or weight data for BMI were missing or implausible for 4 respondents.

**Table 2:** Social media platform usage among 101 adolescents of color in a mixed methods study (2021).

	Ever used platform (N=101), n (%)	Most used platform (N=93), n (%)	Preferred platform for advice on health behaviors (N=101), n (%)	Preferred platform to connect with others on health behaviors (N=100), n (%)
TikTok	80 (80.2)	38 (40.9)	56 (55.4)	44 (44.0)
Instagram	72 (71.3)	16 (17.2)	17 (16.8)	22 (22.0)
YouTube	70 (69.3)	7 (7.5)	2 (2.0)	4 (4.0)
Snapchat	65 (64.4)	11 (11.8)	4 (4.0)	7 (7.0)
Twitter	41 (40.6)	8 (8.6)	10 (9.9)	8 (8.0)
Pinterest	35 (34.7)	3 (3.2)	N/A^a^	N/A^a^
Facebook	32 (31.7)	6 (6.5)	6 (5.9)	10 (10.0)
Reddit	17 (16.8)	2 (2.2)	1 (1.0)	0 (0.0)
Tumblr	3 (2.97)	0 (0.0)	0 (0.0)	1 (1.0)
Other or none	N/A	2 (2.2)	5 (5.0)	4 (4.0)

^a^N/A: Not applicable; Pinterest was not offered as a survey choice for this question.

Compared to males, female adolescents were less likely to engage in 60 minutes or more of moderate to vigorous physical activity daily (16.3% vs 38.8%; *P*=.01) and consumed more daily servings of SSBs (mean 2.0, SD 1.4 vs mean 1.4, SD 1.3; *P*=.04; results not shown in tables). No other significant differences were found between male and female participants in terms of age, social media usage frequency, screen time, BMI, fruit and vegetable intake, or sleep measures. Participants with overweight or obesity were significantly more likely than those with healthy weight or underweight to report consuming more than one daily serving of SSBs (66.7% vs 45.5%; *P*=.04; data not shown in tables). No other significant differences in health behaviors by weight status were observed. We were unable to assess whether weight status or health behaviors varied by frequent social media usage due to an insufficient number of respondents (n=12) reporting usage frequency of once per day or less (Table 1).

Among the 93 participants with data on a single most-used platform (TikTok, Instagram, Snapchat, Twitter, or all other platforms combined), a significant difference was found in the distribution of most-used social media platform by gender (*P*=.03). Among males, TikTok was the most-used platform for 40.0% of respondents, followed by Instagram (22.2%) and Twitter (15.6%). Only 4.4% of males reported Snapchat as their most-used platform. Female respondents also reported TikTok as their most-used platform (41.3%), followed by Snapchat (19.6%) and Instagram (13.0%), with only 2.2% of females reporting Twitter as their most-used platform. The distributions of the most-used social media platform, preferred platform to receive health advice, and preferred platform to connect with others did not vary significantly by weight status (data not shown in tables).

### Focus Group Results

#### Overview

A total of 20 adolescents of color ages 14-18 years (80% female) from 8 youth-based settings participated in 1 of 3 web-based focus groups for the qualitative portion of this study. Using a directed content analysis approach, 4 themes were identified and coded based on the focus group guide: preferred social media platform, purpose of platform use, satisfaction and engagement with platform, and preferences for healthy weight management content and delivery. These themes are summarized in Table 3 and presented alongside illustrative quotes.

**Table 3:** Illustrative quotes by theme from focus groups with 20 adolescents of color in a mixed methods social media study (2021).

Themes			Illustrative quotes
**Preferred social medial platform (overall)**			
	TikTok	“TikTok [has] a lot of versatility to it…there’s more than one thing you can do…you can watch videos, or you can make them, and it's just fun to do.” “TikTok would be my favorite app… you can meet new people through comments and stuff like that, so I think that's fun about it.” “TikTok literally has everything you can search… all the other social media [platforms] keep up with people's [lives] instead of learning stuff.”	
	Instagram	“I use Instagram because I like seeing what's going on in the world.” “There's like a lot of pages, especially on Instagram…that show appreciation for having darker skin or…having a certain type of body.”	
**Platform preference use by purpose or function**			
	Variety and entertainment	“[TikTok] is just nonstop content…literally never ending…it'll just make you laugh the whole time.” “Tik tok literally has everything…you can search for [dance, art, health]…for other social media platforms, you [see] other people’s loves.”	
	New content and information	“[On TikTok] there's always something new…give you new ideas.” “If you ever want to learn something…there's multiple creators [on TikTok] who are specifically posting content about that topic.” “TikTok has taught me…a lot of stuff that they don't teach you in school.” “If I can't find it [on TikTok]…then I will go on on YouTube and find it because YouTube has a lot of various options as well.”	
	Messaging and communication	“I use Instagram a lot to message my friends and family members because we all use Instagram to message each other.” “[For Snapchat], you can communicate a message directly with friends, and then you can post stories to it as well.” “I usually use Snapchat to…connect with friends and see what my friends are doing…on a daily basis.” “Snapchat [is] the way I can talk to people without having their contact information.”	
	Maintaining real-life connections	“Snapchats [are] the main communication when I talk to my friends.” “I use Facebook to reach out to people, mostly to stay connected with my family, my friends, because it's very easy to tag people.”	
**Satisfaction and engagement with platform**			
	TikTok	“TikTok would be my favorite app… you can meet new people through comments and stuff like that, so I think that's fun about it.” “Time goes by so fast and you're laughing…you just keep scrolling and clicking…it kind of like changes your mood.”	
**Preferences for health weight intervention content and delivery**			
	TikTok as platform for health content; Instagram for live engagement	“I like to follow a lot of health content [on TikTok].” “I prefer TikTok…they do help videos [that you can] incorporate with your life and…the things you do daily.” “Instagram and TikTok are the ones to go to because those are the ones that platforms that spread the most information.” “Instagram Live is…more popular…. getting questions from [the] audience really helps because a lot of people [can] relate… … they're asking different questions that you might have thought of, or you haven't [thought of].” “Instagram…[is] a good one would where everyone could interact.”	
	Content on physical and mental health	“I think that people could benefit [from nutrition and exercise content] because we're young…and it's easier for us to lose weight…when you're an adult, it's a lot harder to lose weight. So you can start making a change now so you can be better off in the future.” “[Content on] physical and mental health….because you got to be mentally motivated for a physical kind of thing.” “Promoting mental health more…nobody actually talks about their emotions [or] problems, they keep it inside and hide it.” “[Content on] my mental health and also my physical health, like my diet and my weight in particular. I'm very conscious about it.”	
	Peer messengers and trusted sources	“[On TikTok] we see…people who work in the medical field…who have training.” “[On] Tik Tok…you can follow certain creators for the content that you want to see like…like an OB/GYN I know…I follow [her].” “[I want to hear from] people our age…because they know what we are going through and have the same mindset.” “[It] felt good, very good to see somebody like in your shoes, and then see them get better, because it's very encouraging.” “I know I'm like not the only one… finding people that are like relatable to me would help...”	
	Relatable posts and users on healthy eating and physical activity	“[It’s] very good to see somebody in your shoes, and then see them get better, because it's very encouraging…[you realize] it's not just me, I could do it too.” “…People relate more when they see people [who] look like them talking about [health information]…people of all different races and all different body shapes, all different sexes…”	
	Frequent microhealth tips	“I like when people give out a little tip… little steps to help you get through the day…those are really helpful.” “if you keep seeing it over and over…it's going to motivate you even more.” “…it helps when the [creator] is consistent or the page is consistent with new content.”	
	Design methods to enhance engagement	“try to include [the post] with new songs today…songs that we all know…it [makes it] more pleasing to look at instead of somebody's just talking to you about it.”	

#### Preferred Social Media Platform (Overall)

TikTok emerged among focus group participants as the overall preferred platform of choice generally and with respect to health content. Most participants preferred TikTok due to its versatility, tailored content for the individual user, and options for multiuse engagement (watching, making videos, and interacting with other users through comments, liking, sharing, and dueting). The majority of participants also described initiating use or engaging in more frequent use of TikTok as a result of being isolated from their peers during the first year of the COVID-19 pandemic. Instagram emerged as the second most preferred platform with ease of being able to visually share information and updates, follow and engage with people outside of one’s personal network (eg, influencers and celebrities), and connect with real-life network members (eg, family members, friends, and classmates).

#### Platform Preference by Purpose or Function

Participants discussed using multiple social media platforms for different purposes. With respect to obtaining new content or information and entertainment, TikTok emerged as the preferred platform. With respect to frequent (eg, daily and weekly) messaging and engaging in conversation with others, including interfacing with new individuals or accounts, Snapchat and Instagram were identified as the preferred platforms. In terms of maintaining real-life connections (eg, longer-term updates) with family members and friends, participants identified using Snapchat, Instagram, and Facebook.

#### Satisfaction and Engagement With Platforms

The majority of focus group participants discussed TikTok as yielding the highest amount of satisfaction and engagement. Participants reported that often learning or being exposed to something uplifting, positive, or humorous (resulting in improved mood) on TikTok and that this platform offered a variety of content that was constantly tailored to user preferences. Participants also valued TikTok as a platform for its novelty (“always something new to see”) and relatability (“can always find something [or someone] to relate to”). The second most preferred platform was Instagram, where participants enjoyed viewing updates, viewing inspirational content, and seeing representation of other peers.

#### Preferences for Weight Management Content and Delivery

Key suggestions and preferences for healthy weight management content and delivery via social media that participants voiced included content that addressed the intersection of mental and physical health (eg, body image and the types of strategies to engage in healthy eating and physical activity that also improve mental well-being); peer messengers and information from trusted sources (eg, health care providers and health experts); posts or content that were relatable (eg, information, strategies, and stories from people going through similar experiences); and consistency and frequency in posting content on small, attainable behavioral goals (eg, frequent micro tips on healthy eating or exercise routines). Participants’ views on the ideal source for health-related content were mixed; some participants wanted to see content from health professionals only, whereas other participants preferred to view content from peers with the same everyday, lived experiences.

## Discussion

### Principal Findings

This mixed methods study is the first to our knowledge to examine social media platform use, design, and content preferences for social media–delivered weight management interventions among adolescents of color. Participants in our study reported most frequently using TikTok, followed by Instagram, Snapchat, and Twitter, and preference for platform varied by purpose of use. Participants reporting using TikTok primarily for entertainment, consumption, and creation of content, Snapchat and Instagram for frequent communication or messaging, and a variety of platforms (Snapchat, Instagram, and Facebook) to stay connected with family, friends, and peers. These findings are similar to a small pilot study conducted among adolescent girls aged 12-18 years who reported using Snapchat and Instagram as ways to communicate and stay connected with friends [15].

In concordance with recent trends on social media use among US teens ages 13-17 years from a 2022 Pew Research Center report, TikTok was the most popular social media platform reported by our sample of adolescents of color aged 14-18 years, followed closely by Instagram and Snapchat [14]. These patterns were reflected in both the study’s survey and focus group data. Findings from our study are also in line with 2022 Pew data indicating higher shares of Black and Hispanic teens reporting usage of TikTok, Instagram, and Twitter compared with White teens. Focus group data from our study also support prior research findings that while adolescents are often exposed to health information via social media, they more frequently turn to websites versus social media when proactively searching for health information and are aware of the need to evaluate the accuracy and trustworthiness of health information obtained on the internet [19-21].

Importantly, our study added to the literature by examining adolescents’ preferred platform by function (information vs connection) in the context of a healthy weight management intervention and solicited open-ended input from adolescents of color on their preferences for health intervention content, design, and delivery. Quantitative and qualitative data from this study highlighted TikTok as one of the top preferred platforms of choice for learning new information on healthy weight management, and TikTok and Instagram as preferred platforms for connecting with others (eg, live discussion, commenting, and supporting) on healthy weight management behavioral changes. This study additionally identified adolescents’ specific healthy weight intervention design preferences, such as trusted sources for health information (health professionals and peers), consistency and high frequency of content exposure, importance of being able to relate to others through content (creator or story is one that they can identify with) or connection (engaging with other viewers going through similar experiences), and priority topics they would find engaging and relevant (intersection of physical and mental health).

Compared to national estimates, the prevalence of certain health behaviors among our target sample of adolescents of color (majority Latine) differed; 44% of our study sample reported consuming 5 or more servings of fruits and vegetables daily and 39% reported sleeping 8 or more hours per night compared to 15% and 26% of adolescents from the 2017 Youth Risk Behavior Surveillance Survey, respectively [22]. With respect to interest in intervention content on physical fitness, diet, and mental health, our study findings were consistent with a prior study on social media use and mental well-being among participants aged 14-22 years that reported fitness, nutrition, stress, anxiety, and depression as the top 5 topics searched on social media platforms [23].

To date, there is a lack of research on healthy weight management or obesity interventions delivered via social media among adolescents in the United States. A few studies have examined the efficacy of mobile health interventions targeting diet, physical activity, and BMI among adolescents with promising improvements in certain health behaviors, though the vast majority of these interventions are app or text-based, are not delivered via social media, and lack adequate representation of participants of color [24-26]. One pilot intervention study targeting weight-related behaviors among primarily White adolescents aged 14-18 years incorporated social media (Facebook) as an intervention component to enhance engagement [27]. Participants in the aforementioned study experienced an average increase in steps, though the majority of participants reported preferring other social media platforms such as Instagram over Facebook for intervention purposes. This body of literature combined with findings from this study highlights gaps in the field in leveraging social media as an intervention modality to promote healthy eating and physical activity among adolescents, particularly those of color.

Given the high rates of social media use among adolescents and the prevalence and fast spread of inaccurate health information on social media, the following design considerations, based on our results, may be helpful to guide the development of social media–delivered interventions targeting healthy weight management behaviors among adolescents of color [28]. These include designing content that can be readily disseminated via multiple platforms to reach multiple audiences; matching intervention activities with platforms that are most suited to meet the activity’s purpose (eg, knowledge transfer, connect with others, and sustain motivation); collaborating with adolescent peer leaders to co-design and deliver content to enhance engagement and relatability of content; incorporating frequent microhealth tips; and integrating discussion of physical and mental health topics.

Study strengths include the recruitment of adolescents of color who experience disproportionately higher rates of obesity and are underrepresented in health research and the use of quantitative survey and qualitative focus group methodology to understand patterns and preferences for a social media–based intervention targeting healthy weight management behaviors. Qualitative data provided additional context of the reasons behind participants’ preferences of certain social media platforms over others, why certain platforms were preferred for a social media–based intervention targeting healthy weight management, and what respondents felt should be included in such an intervention. Study limitations include a relatively small sample size of 101 survey respondents and 20 focus group respondents (compared to larger polls or cohort studies), where most focus group respondents were female (thus limiting the male perspective); cross-sectional assessments, which limit our ability to examine changes over time; the use of self-reported measures, which may be subject to recall and social desirability bias; selection bias (eg, participants with greater interest in healthy eating and physical fitness may be more likely to participate in this study than those who did not); and convenience sampling, which resulted in a majority Latine sample recruited from Massachusetts and California, thus limiting generalizability of study findings.

### Conclusions

Given the near ubiquity and high prevalence of social media use among adolescents and adolescents of color, a social media–delivered intervention has a high potential to reach and engage adolescents of color in healthy weight management behaviors. Findings from this study, along with further partnership with adolescent peer leaders, can be used to begin to inform the choice of platform and development of content and strategies for future social media–delivered interventions for youths of color and subsequent efficacy trials of intervention approaches tailored for this population.

## Appendix

Multimedia Appendix 1 Focus group guide for 20 adolescents of color participating in a mixed methods pilot social media study.

## Abbreviations

BGC: Massachusetts Alliance of Boys & Girls Clubs
SSB: sugar-sweetened beverage
