# Supplementary material for: Exploring Social Media Preferences for Healthy Weight Management Interventions Among Adolescents of Color: Mixed Methods Study
Source: JMIR Pediatr Parent. 2023 May 8;6:e43961. doi: 10.2196/43961 (PMC10203922; doi:10.2196/43961)
Supplement: Multimedia Appendix 1 [file pediatrics_v6i1e43961_app1.docx]

**Questions and Discussion**

1. How many of you have a phone and use social media?
2. ______/________
3. What social media platforms do you use the most? Why do you prefer a certain social media platform over others?
4. Tell me more about why you use X platform. (e.g. connecting with friends and family, finding information, following celebrities, learning a skill, etc)
5. How often do you see health-related content on social media? What are some of the thoughts you have about the content you see related to health on social media?
6. Do you feel like these resources are relatable to you?
7. What is the top challenge or most pressing thing on your mind when you think about your health?
8. Why is that?
9. What kinds of things would like to see happen to alleviate/help this?
10. What is your topmost challenge related to your emotional and mental health?
11. What kinds of things would like to see happen to alleviate/help this?
12. Can you think of any mental health resources for teens your age? Do you relate to these resources? Are they helpful to you?
13. Tell me about a time you wanted to find health information online. How did you decide where to look?
14. What are your thoughts on the resources you found?
15. Some people have said that one way to improve teens health is to provide healthy eating and physical activity program through social media. What are your thoughts/feelings about that?
16. What would have to be included in a social media healthy eating and physical activity program for you to want to participate?
17. Tell me about things in your life not related to your health that concern you. What kinds of things would like to see happen to alleviate/help this?

**Program Engagement**

How did you hear about this group? How did each of you decide to participate? What encouraged you to participate?

1. What ideas do you have for making future health programs on social media more fun and exciting? On what social media platform would you like to see health programs?

- What would make it interesting?
- What would the program have to incorporate for you to participate?
